# *Yersinia pestis* strains of ancient phylogenetic branch 0.ANT are widely spread in the high-mountain plague foci of Kyrgyzstan

**DOI:** 10.1371/journal.pone.0187230

**Published:** 2017-10-26

**Authors:** Galina A. Eroshenko, Nikita Yu Nosov, Yaroslav M. Krasnov, Yevgeny G. Oglodin, Lyubov M. Kukleva, Natalia P. Guseva, Alexander A. Kuznetsov, Sabyrzhan T. Abdikarimov, Aigul K. Dzhaparova, Vladimir V. Kutyrev

**Affiliations:** 1 Russian Research Anti-Plague Institute “Microbe”, Federal Service for Surveillance in the Sphere of Consumers Rights Protection and Human Welfare, Saratov, Russian Federation; 2 Center for Quarantine and Particularly Dangerous Infections, Department of Disease Prevention of the Ministry of Health of the Kyrgyz Republic, Bishkek, the Kyrgyz Republic; University of Florence, ITALY

## Abstract

Fifty six *Yersinia pestis* strains, isolated over the period of more than 50 years in three high-mountain foci of Kyrgyzstan (Tien Shan, Alai, and Talas), have been characterized by means of PCR and single nucleotide polymorphism (SNP) typing methods. Seven of these strains were also characterized by means of whole genome sequencing and genome-wide SNP phylogenetic analysis. It was found that forty two strains belong to 0.ANT2, 0.ANT3 and 0.ANT5 phylogenetic branches. From these, strains of 0.ANT2 and 0.ANT3 branches were earlier detected in China only, whereas 0.ANT5 phylogenetic branch was identified for *Y*. *pestis* phylogeny for the first time. According to the results of genome-wide SNP analysis, 0.ANT5 strains are ones of the most closely related to *Y*. *pestis* strain responsible for the Justinianic Plague. We have also found out that four of the studied strains belong to the phylogenetic branch 2.MED1, and ten strains from Talas high-mountain focus belong to the phylogenetic branch 0.PE4 (sub-branch 0.PE4t). Established diversity of *Y*. *pestis* strains and extensive dissemination of the strains pertaining to the 0.ANT branch confirm the antiquity of the mentioned above plague foci and suggest that strains of the 0.ANT branch, which serve as precursors for all highly virulent *Y*. *pestis* strains, had their origin in the Tien Shan mountains.

## Introduction

*Yersinia pestis* is the etiological agent of plague, particularly dangerous infectious disease. Extremely high virulence, potentially high level of the threat to the public healthcare, and dramatic historical experience are among the reasons for the intensive research of *Y*. *pestis* using the high resolution molecular-genetic technologies. In accordance with classification used in the CIS countries (Commonwealth of Independent States), *Y*. *pestis* species includes five subspecies (*ssp*.): *Y*. *pestis ssp*. *pestis* (the main subspecies; includes the strains that are highly virulent and epidemically significant), and four non-main subspecies–*Y*. *pestis ssp*. *caucasica*, *Y*. *pestis ssp*. *altaica*, *Y*. *pestis ssp*. *hissarica*, *Y*. *pestis ssp*. *ulegeica* [1]. The strains of non-main subspecies are often called “pestoides”, and some of them can cause sporadic cases of plague in humans. *Y*. *pestis* strains of the main subspecies, in turn, are subdivided into three biovars: *Antiqua*, *Medievalis* and *Orientalis* [2]. Strains of medieval biovar do not reduce nitrates, strains of oriental biovar do not process glycerol, and strains of antique biovar are positive on both characteristics. Recently, M. Achtman *et al*. [3] during the study of *Y*. *pestis* microevolution with molecular genetics methods has found eight populations of strains and recommended to group strains based rather on their molecular characteristics, than biovars. However, we believe that division of strains into biovars does not contradict to the established modern phylogeny of *Y*. *pestis*. But it should be noted that taken alone it offers more limited resolution for phylogenetic placement. Therefore it should be used together with genetic characteristics of the strains. Thus, strains that are not only incapable of reducing nitrates, but also include marker mutation in the *napA* gene belong to medieval biovar (equivalent to phylogenetic branch 2.MED). The inability to reduce nitrates in some strains from other groups has different genetic grounds and, therefore, they do not belong to medieval biovar.

Antique biovar includes ancient branch 0.ANT, which gave rise to other evolutionary branches of antique strains: 1.ANT, 2.ANT, 3.ANT, and 4.ANT. Strains of oriental biovar (branch 1.ORI) eventually originated from 1.ANT, and strains of medieval biovar (branch 2.MED)–from 2.ANT. The strains of non-main subspecies are irrelevant to *Y*. *pestis* biovars. They are at the root of the ancient branch 0, and are classified as 0.PE2-0.PE7 [3–5]. They are closely related to the plague agent ancestor, *Yersinia pseudotuberculosis* [6]. The strains of *ssp*. *caucasica* are equivalent to phylogenetic branch 0.PE2, *ssp*. *altaica* and *ssp*. *hissarica*– 0.PE4, *ssp*. *ulegeica* (the youngest branch among 0.PE strains)–to phylogenetic branch that is not represented in the common branch system identification.

In spite of advances in genomic sequencing of a considerable number of present and ancient *Y*. *pestis* genomes, the key issues, such as the time and place of *Y*. *pestis* origin, the causes and sources of the three historical pandemics, and the human role as the source of epidemics and pandemics, are still under discussion in the scientific literature. For instance, it was assumed that *Y*. *pestis* pathogen appeared 1,500–20,000 years ago [3], apparently in China, from where it was repeatedly imported into Europe through the trade routes up to nineteenth century [4]. However, the identifying of *Y*. *pestis* in the samples from the ancient human burial sites found in Siberia (in the Altai area) demonstrated that the plague-causing bacteria were widely presented through all Eurasia in the Bronze Age, i.e. at least three thousand years before any historical records of plague pandemics. Moreover, these findings have also demonstrated that those ancient plague strains might be probable ancestors of all known *Y*. *pestis* strains [7]. It was also suggested that the plague outbreaks in Europe between 14th-18th centuries during the second plague pandemic did not result from a multiple importations of plague into Europe, but were generated from the ancient plague focus in Europe [8].

Presently, it becomes obvious that comprehensive information on *Y*. *pestis* strains, circulating in the natural foci of the world (especially in the countries located between Europe and China), is necessary for determination of the evolutionary trends and historical dissemination of *Y*. *pestis*. Among those countries is Kyrgyzstan, most of the natural foci of which are located on the border with China. A little data for the *Y*. *pestis* strains circulating through this territory are available, and these data are mainly regarding phenotypical properties of *Y*. *pestis* strains [1].

**Fig 1:**
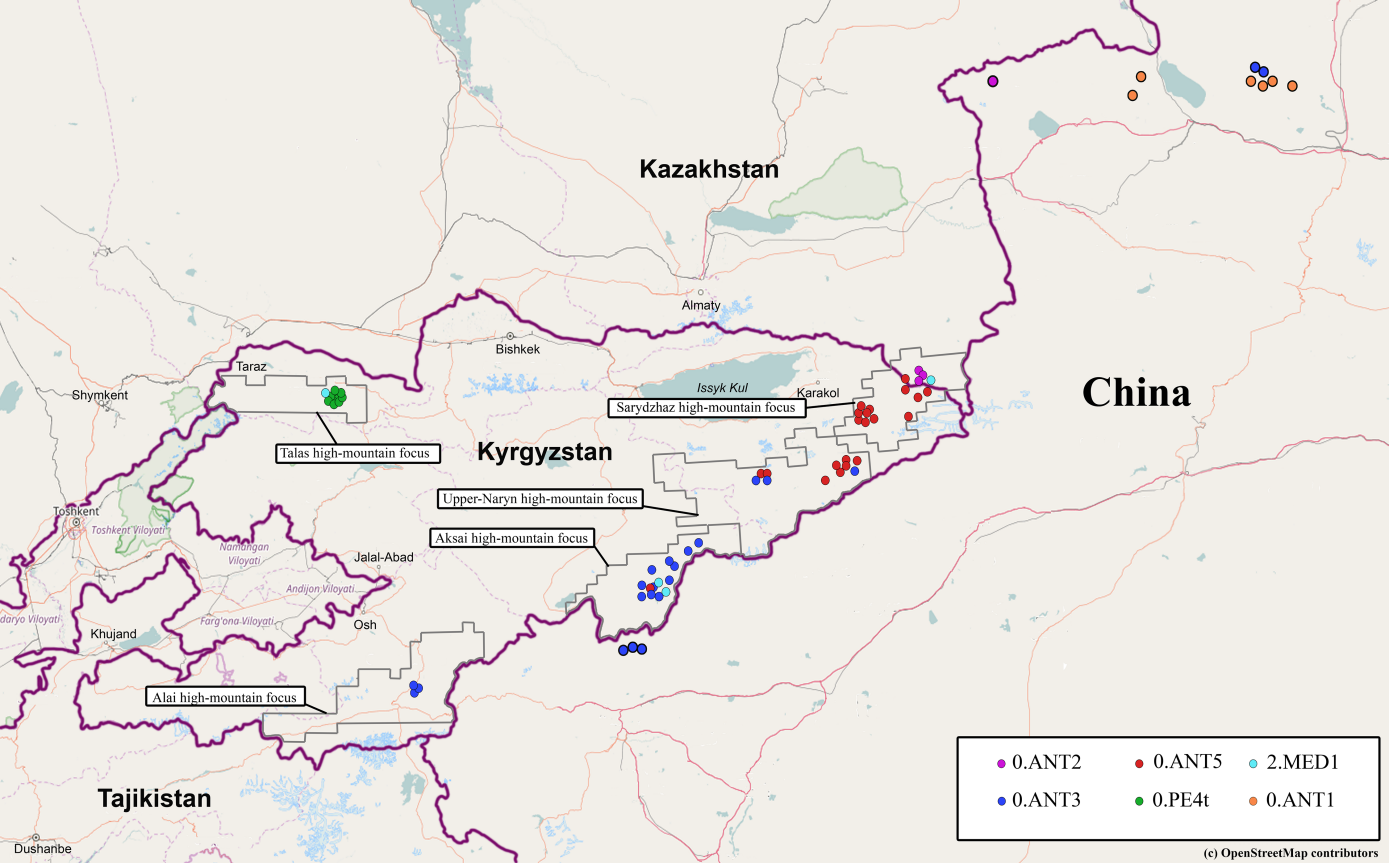
Natural plague foci of Kyrgyzstan and distribution of *Yersinia pestis* strains of different phylogenetic branches across these foci. The geographical location of strains of the 0.ANT1, 0.ANT2 and 0.ANT3 branches in the territory of China are taken from [5]. When preparing this figure a map from OpenStreetMap site was used (www.openstreetmap.org/copyright).

Three high-mountain natural plague foci are located in the mountain area of the Kyrgyz Republic: Tien Shan high-mountain focus (a group of autonomic foci), Alai high-mountain and Talas high-mountain foci (Fig 1). The climate in these foci is sharply continental, and the relief is rugged and mosaic, which determines variable climate conditions, as well as the flora and fauna species variety in certain parts of the foci. It also causes emergence of the micro-foci within these foci.

Enzootic plague territory of the Central Tien Shan is located along the border between Kyrgyzstan and China. The Tien Shan high-mountain focus consists of three autonomous foci: Sarydzhaz, Upper-Naryn, and Aksai. A part of Sarydzhaz high-mountain focus is located in Kazakhstan. The main carrier of the plague infection is the gray marmot, *Marmota baibacina*. In residential communities, situated in the Central Tien Shan region, plague outbreaks emerged on repeated occasions [1].

Alai high-mountain plague focus is located in the south part of the Kyrgyz Republic, in the north-western part of Alai Valley (Fig 1). The main carrier of plague infection in this focus is the long-tailed marmot, *M*. *caudata*. The third plague focus, Talas high-mountain focus is situated in the north-western part of Kyrgyzstan. *Y*. *pestis* strains were isolated here from the long-tailed marmots and from mountain vole, *Alticola argentatus*.

Up to date, intraspecific and phylogenetic appurtenance of *Y*. *pestis* strains from the foci of Kyrgyzstan has not been investigated by means of modern molecular-genetic methods. In this study, we have investigated the collection of *Y*. *pestis* strains, which were isolated in the Kyrgyzstan foci over a period of 50 years, to analyze their properties and phylogenetic relations to the strains, obtained from other regions of the world. Using PCR–and SNP typing of 56 *Y*. *pestis* strains and whole genome sequencing of seven of them, we have found out for the first time that the strains, which belong to the ancient 0.ANT2, 0.ANT3 and 0.ANT5 phylogenetic branches are widely presented in the investigated foci. Strains of 0.PE4t and 2.MED1 branches are also identified there. Diversity of *Y*. *pestis* strains and extensive distribution of the strains which belong to the 0.ANT branch testify to the antiquity of the mentioned above plague foci and suggest that strains of the 0.ANT branch, which serve as precursors for all highly virulent *Y*. *pestis* strains, had their origin in the Tien Shan mountains.

## Materials and methods

### *Yersinia pestis* strains

*Y*. *pestis* strains from three high-mountain plague foci of Kyrgyzstan (Tien Shan, Alai, and Talas) were investigated in this study. The strains were isolated mainly from marmots–*Marmota baibacina* and *M*. *caudata* and their fleas (S1 Table). All strains were received from the State collection of pathogenic bacteria (Russian research anti-plague institute “Microbe”), where they were stored in lyophilized form. Cultivation of strains and analysis of their biochemical properties were performed in accordance with the standard laboratory diagnostic protocols [9].

### PCR- and SNP typing

To determine intraspecific and phylogenetic relations of the studied *Y*. *pestis* strains we have used a system of molecular typing based on PCR and SNP assays. PCR assay was performed using indel mutations that served as markers for *Y*. *pestis* subspecies and biovars [6, 10–12].

Intraspecific differentiation was based on the size of amplifiable loci or presence/absence of these loci (S2A Table). *Y*. *pestis* strains typical to different intraspecific populations were used as a positive control when conducting PCR to avoid false negative results.

To differentiate the strains of the main subspecies, *ssp*. *pestis*, mutations in *gptB-yoaE* and *ilvB-ilvN* loci were identified, in which strains of this subspecies had 89 bp and 45 bp deletions, respectively (S2A Table). Two target sequences, MED24 and glpD, were used to differentiate between biovars of *Y*. *pestis*. To differentiate *Y*. *pestis* strains of two phylogenetic branches (branch 1 and 2), the DNA targets designated as 1.ANT/1.ORI and 2.ANT/2.MED were detected. In the first one, the strains of the branch 1 contain a part of cusφ phage sequence, which is absent in other *Y*. *pestis* strains. In the second one, 2.ANT/2.MED, the strains of the branch 2 contain a marker deletion of 70 bp size. 4.ANT strains were identified using the region of pTP33 plasmid, which is found in all strains of this branch.

To identify the relation of *Y*. *pestis* strains to certain phylogenetic populations marker SNPs were found (S2B Table). The search of the SNPs was conducted with Wombac v2.1 software (https://github.com/tseemann/wombac), based on the comparison between whole genome sequences of *Y*. *pestis* strains, representing different phylogenetic branches. For SNP typing, each SNP was amplified in PCR, then sequenced and aligned against the genome of the reference strain *Y*. *pestis* CO92 (accession number NC_003143) to recognize single nucleotide substitutions. Sequencing of PCR fragments was performed using Genetic Analyzer ABI PRISM 3500XL (Applied Biosystems) in accordance with the manufacturer’s guidelines. For each phylogenetic branch, one or two marker SNP’s were selected.

### Whole genome sequencing

Whole genome sequencing of *Y*. *pestis* strains was performed using Ion PGM system (Life technologies). For the processing of the obtained data and raw short-read sequences assembling *de novo* Ion Torrent Suite software package 5.2 and Newbler gsAssembler 2.6 were applied. The sequence reads were assembled into genomes, resulting in average coverage per genome of 70.8-fold and an average genome assembly size of 4.61 Mb. The detailed description of assembly results is provided in S3 Table. The search of the unique genes in the genome of the sequenced strains was performed by mapping of the received reads on the genome of the reference strain CO92 using software SeqMan (DNASTAR Lasergene 11.2). Not mapped reads were assembled in contigs with SPAdes GenomeAssembler 3.10 and analyzed with the BLAST algorithm in order to find new genes, specific to the studied strains.

### Phylogenetic analysis

Core SNPs were identified by aligning contigs of *Y*. *pestis* strains to CO92 genome, using Wombac v2.1, and after that, 28 homoplastic SNPs were excluded [4]. To the resulting SNPs matrix 157 core genome SNPs, identified in the Altenerding genome (Justinianic Plague) [13, 14], were added (S4 and S5 Tables). A maximum likelihood tree was constructed using the software PHYML with the HKY85 model and 500 bootstrap replications.

## Results

Fifty six *Y*. *pestis* strains were investigated in this study, including 42 strains obtained from Tien Shan high-mountain focus (16 strains from Sarydzhaz focus, 12 strains from Upper-Naryn focus, and 14 strains from Aksai focus), three strains from Alai high-mountain focus, and 11 strains from Talas high-mountain focus (S1 Table, Fig 1). According to the differential biochemical characteristics, all 45 studied strains of *Y*. *pestis* from Tien Shan and Alai high-mountain foci belong to the main subspecies (*ssp*. *pestis*) of the plague bacterium. From those, three strains do not reduce nitrates, which suggests that they belong to medieval biovar; other strains are classified as relative to the antique biovar. Out of 11 *Y*. *pestis* strains from Talas high-mountain focus, one strain belongs to medieval biovar, and the other 10 strains are classed as non-main subspecies of *Y*. *pestis*. All analyzed strains have three residential plasmids (pCD1, pMT1, pPCP1) and do not contain any additional plasmids.

### PCR–and SNP typing

In order to specify intraspecific and phylogenetic relations of *Y*. *pestis* strains obtained from the foci of Kyrgyzstan, we have conducted molecular-genetic analysis using PCR- and SNP typing. For this, we used previously developed system of molecular typing, based on PCR and SNP assays [6, 10, 12]. The PCR assay was conducted using the indel mutations, which are the markers for *Y*. *pestis* subspecies and biovars, while SNP typing was performed using SNPs, which are the markers for the certain phylogenetic branches.

All 45 investigated by means of PCR typing *Y*. *pestis* strains from Tien Shan and Alai high-mountain foci belong to the main (*ssp*. *pestis*) subspecies. They contain specific for this subspecies deletions of 89 and 45 bp in *gptB-yoaE* and *ilvB-ilvN* loci (S2 Table). Three strains out of 45 contain 24 bp deletion in MED24 locus, which together with the absence of nitrate reduction indicates that they belong to 2.MED branch. Other 42 studied strains belong to antique biovar because they lack both, this mutation and 93 bp deletion in *glpD* gene, which is specific for oriental biovar.

Further differentiation of the predominant strains of antique biovar circulating in the Tien Shan and Alai high-mountain foci, according to their appurtenance to the 0.ANT, 1.ANT, 2.ANT, and 4.ANT branches was carried out using 1.ANT/1.ORI, 2.ANT/2.MED and 4.ANT DNA targets. It was stated that 42 studied strains did not contain sequence of cusφ phage, marker for strains of 1.ANT (and 1.ORI) branch, as well as that they did not contain 2.ANT (and 2.MED) marker deletion of 70 bp length. Sequence of pTP33 plasmid, marker for 4.ANT, was also absent. Hence, strains of antique biovar from the foci of Kyrgyzstan do not relate to 1.ANT, 2.ANT, 4.ANT branches, and can only belong to 0.ANT or 3.ANT.

For identification of strains, which belong to the 0.ANT1, 0.ANT2, 0.ANT3 branches, or to the 3.ANT1 and 3.ANT2 branches, relevant marker SNPs were selected (S2B Table). The marker SNPs for the 3.ANT1 in the YPO2499 locus, and the marker SNPs for the 3.ANT2 in the YPO1708 locus were not found. Therefore, all 42 strains of antique biovar from Tien Shan and Alai high-mountain foci must belong only to the ancient branch 0.ANT. It was suggested that the strains of 0.ANT branch served as the etiological agent of the first plague pandemic—Plague of Justinian (541–543 AD) [13, 14]. Presently, strains of 0.ANT branch (0.ANT1, 0.ANT2, and 0.ANT3) are circulating in the territory of China. In order to determine if the studied strains from Kyrgyzstan belong to these known populations, marker SNPs for 0.ANT1 (in YPO1404 and YPO2011 loci), 0.ANT2 (in YPO400 and YPO1545 loci) and 0.ANT3 (in YPO1758 locus) were used. No strains of 0.ANT1 branch among those from Kyrgyzstan were detected. However, we have found the strains of 0.ANT2 and 0.ANT3 branches among them. Strains of 0.ANT2 were found only in Sarydzhaz high-mountain focus (3 strains out of 16). No strains of 0.ANT2 were identified in the Upper-Naryn, Aksai, Alai, and Talas high-mountain foci (Fig 1). On the contrary, strains of 0.ANT3 branch were not detected in Sarydzhaz focus, but were found in Upper-Naryn, Aksai and Alai foci. In the Upper-Naryn focus, 3 out of 12 strains were identified as 0.ANT3; in Aksai focus, 11 out of 14 strains were identified as 0.ANT3, and in Alai focus, all the three strains were identified as 0.ANT3.

Twenty two strains of 0.ANT branch have not been identified by the marker SNPs, specific for 0.ANT1, 0.ANT2, and 0.ANT3, which suggests that they belong to another 0.ANT population, not exemplified earlier in the global phylogenetic diversity of *Y*. *pestis* strains. To determine phylogenetic relation of those strains, the whole genome sequencing was performed for two strains: *Y*. *pestis* A-1691 and A-1836 from Sarydzhaz high-mountain focus (see below).

Three of 45 strains from the Tien Shan and Alai high-mountain foci were related to 2.MED branch, as they possess marker deletion of 24 bp length in the MED24 locus, characteristic of such strains. In order to determine their phylogenetic position, marker SNPs specific for the 2.MED1 (in YPO2744 locus), 2.MED2 (in YPO1299 locus), and 2.MED3 (in YPO0652 locus) were selected (S2B Table). It was established that these three strains (one from the Upper-Naryn high-mountain focus and two from the Aksai high-mountain focus) belong to 2.MED1 branch (Fig 1). The strains of this branch are mainly circulating in the plague foci of CIS, Iran, and China.

Among the 11 investigated strains from Talas high-mountain focus, 10 strains are related to 0.PE4 branch of the non-main subspecies, and one strain–to 2.MED1 branch (Fig 1, S1 Table). Thus, out of 56 strains obtained from Kyrgyzstan and investigated by PCR–and SNP typing assays, 42 strains refer to the 0.ANT branch, 4 strains refer to the 2.MED1 branch, and 10 strains–to 0.PE4 branch.

### Whole genome sequencing

Totally, we have sequenced seven strains. Among them, 2 strains were from Sarydzhaz focus, 1 strain from Aksai focus, 1 strain from Alai focus and two strains from Talas focus. Besides, one strain from Hissar high-mountain focus, which is located in Tadjikistan, was sequenced to establish phylogenetic relationships between *Y*. *pestis* strains from these two countries. We have sequenced the following strains: *Y*. *pestis* 231 (accession number in NCBI GenBank JMUF00000000) from the Aksai, A-1836 (LYOL00000000) and A-1691 (LYMQ00000000) from the Sarydzhaz, A-1486 (f LYMP00000000) from the Alai, A-1809 (LYMF00000000) and A-1815 (LPTY00000000) from the Talas high-mountain foci, as well as *Y*. *pestis* A-1249 (LYMN00000000) from Hissar high-mountain focus from Tadzhikistan (S3 Table). These strains were isolated from the main carriers of plague in the foci. Whole genome sequences of these seven strains and of 30 *Y*. *pestis* strains of various phylogenetic branches from NCBI GenBank have been used for the tree diagram construction and for the determination of the relations between the strains from Kyrgyzstan foci and other regions. The tree diagram was created based on the data from the complete genome SNP analysis of these strains. Comparative assessment of genome wide sequences of all 37 strains has revealed 2493 core SNPs.

**Fig 2:**
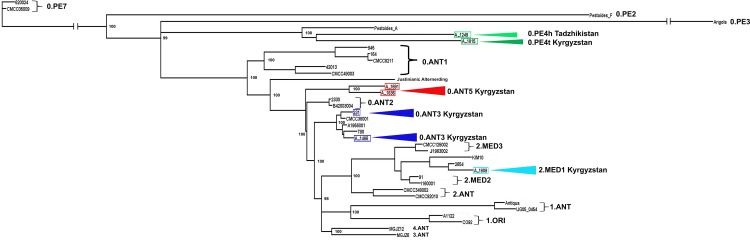
Phylogenetic analysis of *Yersinia pestis* strains from high-mountain foci of Kyrgyzstan based on 2493core SNPs of 37 strains of global origin. Maximum Likelihood tree is constructed using PHYML 3.1 with the HKY85 model and 500 bootstrap replications. Six *Y*. *pestis* genomes from plague foci of Kyrgyzstan and one genome from plague focus of Tajikistan, sequenced in this study, are marked with colored triangles. The names of these strains are circled with colored frames.

Two strains, *Y*. *pestis* 231 from the Aksai and A-1486 from Alai high-mountain foci, are related to the cluster of the 0.ANT3 strains from China (Fig 2). Other two strains, *Y*. *pestis* A-1691 and *Y*. *pestis* A-1836 from the Sarydzhaz high-mountain foci, phylogenetic relations of which we could not specify with SNP typing, made a separate branch on the tree diagram. Those 2 strains differed in the number of unique SNPs from the other 0.ANT strains. We named this newly found branch as the 0.ANT5. The 0.ANT5 strains belong to the phylogenetic node that is one of the closest to *Y* .*pestis* strain of the Justinianic plague (Fig 2).

*Y*. *pestis* A-1809 strain of medieval biovar from Talas high-mountain focus merged with the cluster which is common to *Y*. *pestis* KIM strain from the 2.MED1 phylogenetic branch. One more sequenced *Y*. *pestis* A-1815 strain, representing Talas population of the non-main subspecies strains, merged with the 0.PE4 cluster. It is the most closely related strain to the sequenced A-1249 strain from Hissar high-mountain focus, isolated from Tadzhikistan. To distinguish the strains of the Talas and Hissar populations from other 0.PE4 strains, we have noted the Talas strains as 0.PE4t, and the Hissar strains as 0.PE4h.

In order to determine whether the genomes of *Y*. *pestis* strains from plague foci of Kyrgyzstan include some new genes, we compared the seven sequenced strains with strains from the global collection of NCBI Genbank. We have not found new genes or plasmids that are specific to *Y*. *pestis* strains from this region of the world.

To ascertain whether the 20 of the non-identified by means of PCR- and SNP typing *Y*. *pestis* strains of 0.ANT branch, isolated from Kyrgyzstan foci, belong to the new 0.ANT5 branch (represented by sequenced A-1836 and A-1691 strains), we have selected two marker SNPs, specific for this branch, in the YP0114, and in the YP01105 loci (S2B Table). It was found that all these strains belong to 0.ANT5 branch. The strains of this branch are spread in the Sarydzhaz and the Upper-Naryn high-mountain foci (Fig 1).

Thus, this research has revealed the diversity of *Y*. *pestis* strains in the natural plague foci of Kyrgyzstan related to 0.ANT2, 0.ANT3, 0.ANT5, 2.MED1, and 0.PE4t branches. The strains of the 0.ANT2, 0.ANT5 and 2.MED1 branches are circulating in the Sarydzhaz high-mountain focus, and the strains from the Upper-Naryn high-mountain focus belong to the 0.ANT3 and 0.ANT5 branches. In the Aksai high-mountain focus the 0.ANT3, 0.ANT5 and 2.MED1 branch strains circulate. All the strains from the Alai high-mountain focus belong to the 0.ANT3 branch. The strains isolated from the Talas high-mountain focus belong mainly to the 0.PE4t branch (10 strains) and to the 2.MED1 branch (1 strain). In conclusion, it should be pointed out that we have observed full agreement between the phylogenetic assignments of *Y*. *pestis* strains of all five studied lineages, based on PCR-, SNP typing and whole genome sequencing.

## Discussion

Natural plague foci are found on the majority of the continents. The greatest diversity of *Y*. *pestis* strains is observed in Central-Asian area of natural plague focality. The strains, which belong to the majority of the world phylogenetic branches of *Y*. *pestis* are circulating in this region. The ancient branch 0 is represented with the strains of the 0.PE4 (Russian Federation, China, and Mongolia) and the 0.PE7 (China), as well as with the 0.ANT1, 0.ANT2, and 0.ANT3 (China) strains. The strains of such branches as 2.ANT (Russian Federation, Mongolia, China), 3.ANT (Mongolia, China), 4.ANT (Russian Federation, Mongolia) and 2.MED, including 2.MED1 (mainly, Russian Federation, Kazakhstan, and other CIS countries), 2.MED2, and 2.MED3 (China) are also circulating in this region. Diversity of the strains from natural foci of China and antiquity of the majority of them served as the grounds for the hypothesis that *Y*. *pestis* has originated from China, and that the strains, which caused three historical plague pandemics also had their origin in China, from where they were regularly brought into Europe by Chinese merchants and military commands. However, modern data from studies of the ancient DNAs samples from the antique, medieval and older remains of the victims of plague epidemics do not support this hypothesis. For example, it was found that the oldest from the known *Y*. *pestis* strains were circulating in Siberia more than five thousand years ago, which serves as an argument for plague agent origination in this territory [7]. It was also shown that plague epidemics in Europe during the 14^th^-18^th^ centuries did not result from the multiple introductions of plague into Europe, but were rather associated with long-term persistence of the historical plague focus in Europe [8]. Moreover, it was recently suggested that a wave of plague might have travelled from Europe toward Asia during the Black Dearth time, to later become the source population for contemporary worldwide epidemics [15]. The speculations regarding the origin of *Y*. *pestis* strains of the medieval biovar in the territory of China were not supported as well. In our earlier published study we have demonstrated that the most deeply diverged strains of medieval biovar are persisting in the Central-Caucasian high-mountain plague focus of the Russian Federation. They are named as the 2.MED0 [16].

Based on the presented results for 56 *Y*. *pestis* strains, isolated from the Tien Shan (a group of autonomous foci, Sarydzhaz, Upper-Naryn, and Aksai), the Alai and Talas high-mountain foci, established is the diversity and antiquity of the circulating in the region strains, which belong to the main subspecies, branches 0.ANT2, 0.ANT3, 0.ANT5 and 2.MED1, as well as to the non-main subspecies, branch 0.PE4t. These data demonstrate that the strains of the ancient branches, the 0.ANT2 and the 0.ANT3, are not unique for China. They are distributed in natural foci, located in the Tien Shan mountains including the Tien Shan and Alai high-mountain foci in Kyrgyzstan. Furthermore, the ancient phylogenetic branch, the 0.ANT5, is described, which was not presented in the global phylogeny of *Y*. *pestis* so far. According to the genome-wide SNP analysis, the 0.ANT5 strains are ones of the most closely related to *Y*. *pestis* strain responsible for the Justinianic Plague [13, 14].

Extensive dissemination of the strains of the 0.ANT branch points to the possibility that these strains, which serve as precursors for all highly virulent *Y*. *pestis*, had their origin in the Tien Shan mountains. We can also speculate that several waves of *Y*. *pestis* strains, relating to different branches of plague agent evolution, ran through the territories of these foci at different historical epochs. High-mountain foci of Kyrgyzstan with their mosaic, specific relief, diversity of climatic conditions and species of flora and fauna composition, served as a favorable environment for the settling down of the strains, representing different waves of *Y*. *pestis* outspread.

## Supporting information

S1 Table Strains of *Yersinia pestis* used in this study. (DOCX)

S2 Table DNA targets and primers used for PCR–and SNP typing of *Yersinia pestis* strains. (DOCX)

S3 Table Data production. (XLSX)

S4 Table Strains of *Yersinia pestis* used for phylogenetic analysis. (XLSX)

S5 Table Genomes used for core SNP calling and phylogeny. (XLSX)
