# Supplementary material for: Yersinia pestis strains of ancient phylogenetic branch 0.ANT are widely spread in the high-mountain plague foci of Kyrgyzstan
Source: PLoS One. 2017 Oct 26;12(10):e0187230. doi: 10.1371/journal.pone.0187230 (PMC5658180; doi:10.1371/journal.pone.0187230)
Supplement: S1 Table — (DOCX) [file pone.0187230.s001.docx]

S1 Table. Strains of *Yersinia pestis* used in this study

| N | Strain | Natural plague foci | Year of isolation, source | Phylogenetic branch |
| --- | --- | --- | --- | --- |
| 1 | А-1836 | Sarydzhaz high-mountain focus | 1983, *Marmota baibacina* | 0.ANT5 |
| 2 | A-1710 | –“– | 1971, *Marmota* | 0.ANT2 |
| 3 | A-100 | –“– | 1948, *Oropsylla silantievi* | 0.ANT5 |
| 4 | A-1701 | –“– | 1971, *Marmota* | 0.ANT2 |
| 5 | 915 | –“– | 1948, *Oropsylla silantievi* | 0.ANT5 |
| 6 | 936 | –“– | 1955, *Oropsylla silantievi* | 0.ANT5 |
| 7 | КМ-937 | –“– | 1958, *Marmota baibacina* | 0.ANT5 |
| 8 | 1204 | –“– | 1959, *Marmota baibacina* | 0.ANT5 |
| 9 | 1564 | –“– | 1960, *Marmota baibacina* | 0.ANT2 |
| 10 | A-1837 | –“– | 1983, *Marmota baibacina* | 0.ANT5 |
| 11 | A-1691 | –“– | 1971, fleas of *Marmota baibacina* | 0.ANT5 |
| 12 | 120 | –“– | 1928, patient | 2.MED1 |
| 13 | 14/1646 | –“– | 1959, *Marmota baibacina* | 0.ANT5 |
| 14 | 693 | –“– | 1968, *Oropsylla silantievi* | 0.ANT5 |
| 15 | 694 | –“– | 1968, *Marmota baibacina* | 0.ANT5 |
| 16 | 9/865 | –“– | 1966, *Marmota baibacina* | 0.ANT5 |
| 17 | 333 | Upper-Naryn high-mountain focus | 1961, *Marmota baibacina* | 0.ANT3 |
| 18 | A-1694 | –“– | 1971, fleas of *Marmota baibacina* | 0.ANT3 |
| 19 | 4/692 | –“– | 1966, *Rhodopsylla livetricosa* | 0.ANT5 |
| 20 | 43 | –“– | 1966, *Marmota baibacina* | 0.ANT5 |
| 21 | 262 | –“– | 1967, *Marmota baibacina* | 0.ANT5 |
| 22 | 285 | –“– | 1967, *Marmota baibacina* | 0.ANT5 |
| 23 | 363 | –“– | 1959, *Marmota baibacina* | 0.ANT5 |
| 24 | 680 | –“– | 1968, *Marmota baibacina* | 0.ANT3 |
| 25 | 5М | –“– | 1952, *Oropsylla silantievi* | 0.ANT5 |
| 26 | 88/134 | –“– | 1961, *Rhadinopsylla ventricosa* | 0.ANT5 |
| 27 | 71/1224 | –“– | 1961*, Marmota baibacina* | 0.ANT5 |
| 28 | 72/1458 | –“– | 1962, *Marmota baibacina* | 0.ANT5 |
| 29 | 231 | Aksai high-mountain focus | 1947, *Marmota baibacina* | 0.ANT3 |
| 30 | 934 | –“– | 1955, *Marmota baibacina* | 0.ANT3 |
| 31 | 940 | –“– | 1955, *Marmota baibacina* | 2.MED1 |
| 32 | A-1734 | –“– | 1972, *Marmota baibacina* | 2.MED1 |
| 33 | 1/1-53 | –“– | 1953, *Marmota baibacina* | 0.ANT5 |
| 34 | 1/156 | –“– | 1959, *Marmota baibacina* | 0.ANT3 |
| 35 | 1203 | –“– | 1959, *Rhadinopsylla li ventricosa* | 0.ANT3 |
| 36 | 353 | –“– | 1955, *Marmota baibacina* | 0.ANT3 |
| 37 | 938 | –“– | 1955, *Marmota baibacina* | 0.ANT3 |
| 38 | 235 | –“– | 1947, *Marmota baibacina* | 0.ANT3 |
| 39 | А-744 | –“– | 1963, *Marmota baibacina* | 0.ANT3 |
| 40 | A-1785 | –“– | 1974, *Marmota baibacina* | 0.ANT3 |
| 41 | 339 | –“– | 1956, *Oropsylla sylantievi* | 0.ANT3 |
| 42 | 375 | –“– | 1956, *Ixodes cranulatus* | 0.ANT3 |
| 43 | А-1486 | Alai high-mountain focus | 1966, *Marmota caudata* | 0.ANT3 |
| 44 | A-1487 | –“– | 1966, *Marmota caudata* | 0.ANT3 |
| 45 | 1\220 | –“– | 1966, *Marmota caudata* | 0.ANT3 |
| 46 | А-1805 | Talas high-mountain focus | 1980, *Neopsylla terature,* | 0.PE4t |
| 47 | А-1806 | –“– | 1980, *Amphipsylla primaries* | 0.PE4t |
| 48 | А-1807 | –“– | 1980, *Ceratophyllus caspius* | 0.PE4t |
| 49 | А-1813 | –“– | 1980, *Pectinoctenus nemorosus* | 0.PE4t |
| 50 | А-1814 | –“– | 1980, *Amphipsylla primaries* | 0.PE4t |
| 51 | А-1815 | –“– | 1980, *Ceratophyllus caspius* | 0.PE4t |
| 52 | А-1816 | –“– | 1980, *Amphipsylla primaries* | 0.PE4t |
| 53 | А-1817 | –“– | 1980, *Alticola argenatus* | 0.PE4t |
| 54 | А-1818 | –“– | 1980, *Alticola argenatus* | 0.PE4t |
| 55 | А-1820 | –“– | 1980, *Neopsylla terature* | 0.PE4t |
| 56 | А-1809 | –“– | 1980, *Pectinoctenus nemorosus* | 2.MED1 |
