# Supplementary material for: Yersinia pestis strains of ancient phylogenetic branch 0.ANT are widely spread in the high-mountain plague foci of Kyrgyzstan
Source: PLoS One. 2017 Oct 26;12(10):e0187230. doi: 10.1371/journal.pone.0187230 (PMC5658180; doi:10.1371/journal.pone.0187230)
Supplement: S2 Table — (DOCX) [file pone.0187230.s002.docx]

**PCR typing**

DNA targets, which carry marker mutations, specific to subspecies, biovars, and phylogenetic branches, were used for determination of intraspecific appurtenance of *Y. pestis* strains (S2A Table). The target DNAs were amplified in 25 μl reaction mixture containing 1 x PCR buffer (Invitrogen), 1.5 mM MgCl_2_, 0,2 mM dNTPs (Invitrogen), 10 pM primer (Syntol, Russia) and 1 U Taq DNA polymerase (Invitrogen). Amplification of target DNAs required an initial 5 min denaturation at 94 °C and 35 cycles of 45 sec denaturation at 94 °C, 30 sec annealing at 55-58 °, and a 45 sec extension at 72 °С followed by a final 3 min extension at 72 °С.

Intraspecific differentiation was based on the size of amplifiable loci or presence/absence of these loci (S2A Table). *Y. pestis* strains typical to different intraspecific populations were used as a positive control when conducting PCR to avoid false negative results.

**SNP typing**

In order to identify the appurtenance of *Y. pestis* strains to certain phylogenetic branches, marker SNPs were used (S2B Table). Each SNP was amplified by PCR (95 ºС, 5 min; 35 cycles: 95 ºС – 45 sec, 54 ºC – 40 sec, 72 ºС – 45 sec; 72 ºС – 3 min), sequenced and aligned to reference genome sequence (strain CO92, accession number NC_003143 NCBI GenBank) for search of nucleotide substitutions. One or two SNPs were selected for each relevant branch.

**S2A Table. DNA targets and primers used for intraspecific differentiation of *Y. pestis* strains by PCR**

| **DNA target** | **Localization in the genome of *Y. pestis* С092*** | **Marker character** | **Sequences of primers**  **5’→3’** | **Size of amplifiable locus (bp) or**  **presence + (bp)/ absence –**  **of amplifiable focus** | **Reference** |
| --- | --- | --- | --- | --- | --- |
| *gptB-yoaE* | 2004340-2004639 | Deletion of 89 bp in strains of the main subspecies | S – GCTGCGTATCATTTCACC  As – AATCAAATCTCGCCCAGC | Strains of the main subspecies, 300  Other strains, 389 | [6] |
| *ilvB-ilvN* | 2579684-2580198 | Deletion of 45 bp in strains of the main subspecies | S – AGTGGTCTGCTTCTCTGG  As – CGGCATACACAGAATACC | Strains of the main subspecies, 515  Other strains, 560 | –“– |
| *glpD* | 4421352- 4421370 | Deletion of 93 bp in strains of the main subspecies, oriental biovar | S – CAGAGGAAGGTAACATGGA  As – CTTGGTAAGTGAAGCCTTG | Strains of the oriental biovar, 415  Other strains 508 | [10] |
| MED24 | 996412-996633 | Deletion of 24 bp in strains of the main subspecies, medieval biovar | S – GTATTTTGTGTCACCCC  As – AATGAGACACCGCCAGT | Strains of the medieval biovar 198  Other strains, 222 | –“– |
| 1.ANT/  1.ORI | 2555559 – 2555974 | Presence of cusφ phage sequence in strains of the phylogenetic branches 1.ANT, 1.ORI | S – GGTTCTGCTCTCTGTTTGTC  As – GTAGAGATGTGTTGCCCG | Strains of the phylogenetic branches 1.ANT, 1.ORI +, 416  Other strains – | [12] |
| 2.ANT/  2.MED | 2801285-2801751 | Deletion of 70 bp in strains of the phylogenetic branches 2.ANT. 2.MED | S – AAGACCTTCGCCACCAGA  As – CCAGGATTCGCCGATTCA | Strains of the phylogenetic branches 2.ANT. 2.MED, 397  Other strains, 467 | –“– |
| 4.ANT | 3153-3312 in pTP33 plasmid sequence (accession number No KT020860, NCBI GenBank) | Presence of pTP33 plasmid in strains of the phylogenetic branch 4.ANT | S – CGCCGCCCATTCCGATTTA  As – TCTGGCTTCCCGTTCCTGC | Strains of the phylogenetic branch 4.ANT +, 160  Other strains – | Current study |

* Accession number NC_003143, NCBI GenBank)

**S2B Table. DNA targets and primers used for SNP typing of *Y. pestis* strains**

| **DNA target, position of marker SNP in the genome of *Y.  pestis* С092*** | **Sequences of primers**  **5’→3’** | **Phylogenetic branches** | | | | | | | | | | | | |
| --- | --- | --- | --- | --- | --- | --- | --- | --- | --- | --- | --- | --- | --- | --- |
|  |  | **0.PE4t** | **0.ANT1** | **0.ANT1** | **0.ANT2** | **0.ANT2** | **0.ANT3** | **0.ANT5** | **0.ANT5** | **3.ANT1** | **3.ANT2** | **2.MED1** | **2.MED2** | **2.MED3** |
| YPO0310,  316868 | S – CTACCGAGCACCCTGAACT  As – CCCAGTAACCAAGGCACAT | T | C | C | C | C | C | C | C | C | C | C | C | C |
| YPO1404,  1587996 | S – TTCAGGAGCAACTGGAAGAA  As – TCCGGGCTGAGGTAGAGAT | C | T | C | C | C | C | C | C | C | C | C | C | C |
| YPO2011,  2283467 | S – CAGGCGGTTCATTGCTTT  As – CCGCTTACGCATCGGTATT | T | T | C | T | T | T | T | T | T | T | T | T | T |
| YPO0400,  418282 | S – ATTATGCGGTCGATCTTTTG  As – TGATACCAGAGCCGTCAGTT | G | G | G | T | G | G | G | G | G | G | G | G | G |
| YPO1545,  1760302 | S –CCATGCACGAAGTAGAAATAGG  As – AACGCGAGCTTATCGAACTG | T | T | T | T | G | T | T | T | T | T | T | T | T |
| YPO1758,  2003542 | S – TCAGCCAGCATCGGAATGT  As – CGTCCCAGGTGAAAATGCT | C | C | C | C | C | T | C | C | C | C | C | C | C |
| YP00114,  121618 | S – ATGAACCCAGGTGGTAGCTG  As – ACGGTATCCATTCCGTTGAA | C | C | C | C | C | C | A | C | C | C | C | C | C |
| YP01105,  1249092 | S – CTGCCTGTGGAGAAAATGAA  As – GTACGCCTGTACCCGTATCC | G | G | G | G | G | G | G | A | G | G | G | G | G |
| YPO2499,  2808675 | S – GCCGCGAAGTCAGTACCAG  As – ATTCTGATCATGGCCGGTGT | C | C | C | C | C | C | C | C | A | C | C | C | C |
| YPO1708,  1949381 | S – GGTTTCCAATACGGGCGAT  As – GCTTTGGCGGGACTTCTAA | C | C | C | C | C | C | C | C | C | T | C | C | C |
| YPO2744,  3074598 | S – CCGTATAGCATCGCGGAAC  As – ACCACCTACGCCTTCAACA | C | C | C | C | C | C | C | C | C | C | T | C | C |
| YPO1299,  1458747 | S – GAAACCCAATCGCCGTGAA  As – AGGCATTAACCCACAACGC | C | C | C | C | C | C | C | C | C | C | C | T | C |
| YPO0652,  710418 | S – ACGTGAGCTACGGCCTTTC  As – TCCCCACGTAACCACAGC | C | C | C | C | C | C | C | C | C | C | C | C | T |

* Accession number NC_003143, NCBI GenBank)
